# The role of sleep for memory consolidation: does sleep protect memories from retroactive interference?

**DOI:** 10.3758/s13423-023-02264-8

**Published:** 2023-06-23

**Authors:** Magdalena Abel, Anna T. Nickl, Anna Reßle, Carmen Unger, Karl-Heinz T. Bäuml

**Affiliations:** Department of Experimental Psychology, Regensburg University, Regensburg, Germany

**Keywords:** Sleep, Memory, Memory consolidation, Retroactive interference

## Abstract

Numerous studies suggest that sleep benefits memory. A major theoretical question in this area is however if sleep does so by passively shielding memories from interference that arises during wakefulness or by actively stabilizing and strengthening memories. A key finding by Ellenbogen et al. *Current Biology*, *16*, 1290–1294 (2006a) indicates that sleep can protect memories from retroactive interference, which suggests that sleep plays more than a passive role for memory consolidation. Sample size in this study was however small and subsequent reports in the literature provided mixed results. We therefore conducted an online study via Zoom to replicate Ellenbogen et al. *Current Biology*, *16*, 1290–1294 (2006a). Subjects were asked to study paired associates. After a 12-h delay that included either nocturnal sleep or daytime wakefulness, half of all subjects were asked to study an additional list to elicit retroactive interference. All participants were then asked to complete a memory test for the studied list(s). The results were fully consistent with those reported by Ellenbogen et al. *Current Biology*, *16*, 1290–1294 (2006a). We discuss this successful replication against the background of the mixed literature, with a focus on the possibly critical role of study-design features, like the use of high learning criteria that resulted in performance being at ceiling, or a confound between interference and the length of the retention interval. A collaborative replication effort may be needed to reach a straightfoward answer to the question if sleep protects memories from interference (and under what conditions).

We all spend significant proportions of our lives asleep. Sleep must serve a purpose in order to be adaptive and, indeed, it has been suggested to fulfill more than one critical function (e.g., Frank, 2006; Rechtschaffen, 1998). Regarding human cognition, sleep has been proposed to support memory consolidation, the gradual stabilization of memories after initial encoding. Consistently, numerous studies have reported enhanced recall with an interval of sleep (rather than wakefulness) between encoding and a memory test (e.g., Barrett and Ekstrand, 1972; Jenkins and Dallenbach, 1924). However, this benefit is not only compatible with an active role of sleep for memory consolidation, but can also be explained when assuming a more passive role of sleep.

## Theoretical views on how sleep benefits memory

Central to the more passive view is the potentially differential role of retroactive interference during wake and sleep delay. Retroactive interference arises when, after initial encoding of a target list of items, additional (nontarget) items are encoded, which then compete for recall on a later test. Subjects may study a list of unrelated items (e.g., *tomato*, *iron*) and then study another list of such items (e.g., *butcher*, *couch*). When, at test, subjects are asked to recall the first-list items, second-list items may come to mind. This interference may reduce recall of the first-list items relative to a control condition, in which subjects studied the first list only (for reviews, see Crowder, 1976; Wixted, 2004). Benefits of sleep for memory might arise because additional encoding takes place during wakefulness, but not during sleep. Such shielding from additional encoding may reduce effects of retroactive interference after sleep and thus be at the core of the sleep-associated recall benefit (e.g., Coenen, 2005; Jenkins and Dallenbach, 1924).

In contrast, the active view assumes that enhanced recall after sleep emerges because sleep is directly involved in memory consolidation (e.g., Ekstrand, 1967). Physiological components unique to sleep have been proposed to contribute critically to the strengthening and stabilization of memories. One proposal is that hippocampal reactivations during slow-wave sleep consolidate memories (e.g., Peigneux et al., 2004; Rasch et al., 2007; Rudoy et al., 2009), with specific components of slow-wave sleep, like sleep spindles or sharp-wave ripples, being critically involved (Axmacher et al., 2008; Cox et al., 2012; Schabus et al., 2004). Such reactivations supposedly prompt the gradual transfer of memory representations from hippocampal to neocortical sites for long-term storage (e.g., Diekelmann and Born, 2010). What unites these and similar suggestions is the common assumption of an active role of sleep in memory consolidation (e.g., Rasch and Born, 2013; Stickgold, 2013).

Finally, sleep might also play a permissive role (e.g., Mednick et al., 2011; Wixted, 2004). Following this view, physiological processes unique to sleep are not critically involved in memory consolidation, but instead sleep reduces the demands placed on our cognitive system by cutting us off from ongoing perception and additional learning. Under such conditions, the same consolidation processes that are active during wakefulness (e.g., hippocampal reactivations) may simply be carried out more efficiently. If so, sleep would permit more effective memory consolidation, but would not itself be actively involved (see also Ellenbogen et al., 2006b).

### Does sleep protect memories from retroactive interference?

A key finding arguing against a merely passive role of sleep was reported by Ellenbogen et al. (2006a). The study directly relates to classic consolidation theory as pioneered by Müller and Pilzecker (1900); see also Wixted, 2004), who used retroactive interference to probe the stability of memories, arguing that consolidated memories should be less susceptible.

Ellenbogen et al. (2006a) followed this rationale. Subjects studied an A-B list of paired associates (e.g., *blanket*-*village*). After 12 h filled with nocturnal sleep or daytime wakefulness, half the participants were immediately tested; the stimulus word of each pair (e.g., *blanket*, A) was provided and subjects recalled the appropriate response word (e.g., *village*, B). The other participants studied an additional A-C list of paired associates (e.g., *blanket*-*rubber*) before completing the same test on the first-list items. Benefits of sleep were more pronounced in the presence than in the absence of additional learning. In short, sleep made memories less susceptible to retroactive interference. This finding clearly argues against a passive role of sleep for memory consolidation. If sleep only passively shielded memories from interference accumulating during wakefulness, they should subsequently still show the same susceptibility to experimentally induced retroactive interference, which was not what the data suggested. Ellenbogen et al. (2006a) proposed that hippocampal reactivations during sleep, in coherence with activity in neocortical areas, might stabilize memories and make them more resistant to interference.

Notably, the results reported by Ellenbogen et al. (2006a) arose on the basis of a 2 (sleep vs. wakefulness) x 2 (additional learning absent vs. present) between-subjects design, with only 12 subjects per condition. Despite its strong theoretical implications, the original study with this between-subjects design was never directly replicated with higher statistical power. Single studies in the literature used a between-subjects design as well (for an overview, see Table 1), but most focused on other research questions and used retroactive interference to vary retrieval difficulty. Findings of these subsequent studies were mixed. Bäuml et al. (2014) used semantically categorized item material and varied additional learning across two experiments. The results showed no protective influence of sleep, irrespective of whether materials were practiced via restudy or testing cycles. Schoch et al. (2017) applied neutral and emotional picture materials. The interaction between sleep and additional learning was examined for a subgroup of participants only, but was not significant ($p=.057$). Finally, Petzka et al. (2021) used a temporal-spatial memory task and found sleep-dependent protection from retroactive interference with a 70%, but not with a 50% learning criterion.

**Table 1:** Overview of previous studies that examined whether sleep protects memories from retroactive interference

					Effect size of critical two-way interaction	
Study	Additional learning: Absent vs. present	Sleep vs. wake	n per condition	Full sample size	$\eta _p^2$	*f*
Ellenbogen et al. (2006a)	between	between	12	48	0.11	0.35
Bäuml et al. (2014; practice via restudy)^a^	between	between	36	144	0.001	0.03
Bäuml et al. (2014; practice via testing)	between	between	36	144	0.0003	0.02
Petzka et al. (2021; 70% learning criterion)	between	between	15	60	0.17	0.45
Petzka et al. (2021; 50% learning criterion)	between	between	15	60	0.01	0.10
Schoch et al. (2017; groups with immediate retrieval)	between	between	27-29	112	0.03	0.18
Alger et al. (2012)^b^	within	between	12	36	0.19	0.48
Bailes et al. (2020)	within	between	51/46	97	0.004	0.06
Ellenbogen et al. (2009)	within	between	22/23	45	0.10	0.33
Sheth et al. (2012, Experiment 4)^c^	within	between	12	24	0.004	0.06
Pöhlchen et al. (2020, Experiment 1)	within	within	12	12	0.006	0.08
Pöhlchen et al. (2020, Experiment 2)	within	within	19	19	0.001	0.03

*Note*. Two relevant studies are not shown in this table. A within-subject study by Deliens et al. (2013) is not included because it did not report results for the critical two-way interaction. Results were however contrary to those reported by Ellenbogen et al. (2006a). Moreover, a between-subjects study by Sonni and Spencer (2015) is not included, because this study compared younger and older adults, but results were not reported separately for the two age groups. The pattern of results for younger adults was however consistent with that reported by Ellenbogen et al. (2006a). Additionally, two studies by Diekelmann et al. (2011, 2012) were not included because they did not have control conditions without additional learning. For related work in other non-human species, see also Martin-Ordas and Call (2011) and Brawn et al. (2013)

^a^ In this study, additional learning was varied across two experiments (Experiment 1: additional learning absent; Experiment 2: additional learning present).

^b^ This study included two different napping conditions (10 min, 60 min), resulting in three between-subjects conditions.

^c^ Experiments 1-3 of this study did not include control conditions without additional learning

Other studies in the literature switched to within-participant manipulations of additional learning, with some studied materials being tested before retroactive interference is introduced for the remaining materials. Results of these studies were also mixed. Ellenbogen et al. (2009) used A-B, A-C paired associate learning and replicated Ellenbogen et al.’s (2006a) original finding. Similarly, Alger et al. (2012) asked subjects to learn associations between words and sounds and found that daytime naps involving slow-wave sleep reduced susceptibility to interference. Other studies reported null effects, however. Sheth et al. (2012) conducted several experiments, but the only experiment incorporating a control condition without additional learning showed no sleep-dependent protection from interference. Bailes et al. (2020) conducted a replication of Ellenbogen et al. (2009), finding no evidence of a protective effect of sleep. Finally, again using paired associate learning, Pöhlchen et al. (2020) also found no corresponding evidence. Critically, sample size in most studies was rather small. The most notable exception from this pattern is Bailes et al. (2020), who tested around 50 participants per condition (see Table 1 for sample sizes and effect sizes across studies).

### The present study

To date there is no straight answer on whether sleep does (or does not) protect memories from retroactive interference. Given that this is a key issue for the bigger theoretical question of whether sleep plays merely a passive role for sleep-associated memory consolidation, it is important to get a more definitive answer. In the following, we report a replication study of Ellenbogen et al. (2006a). The replication was aimed at the core finding of the study, and applied the same 2 (12-h sleep vs. 12-h wakefulness) x 2 (additional learning absent vs. present) between-subjects design, albeit with increased sample size.

Our goal was to address two issues. The main issue was whether the key finding by Ellenbogen et al. (2006a) can be replicated. Another issue was whether the relatively large effect sizes reported by Ellenbogen et al. may have been too optimistic. Ellenbogen et al. mentioned effect sizes for single comparisons only: the nonsignificant difference between sleep and wake groups in the absence of retroactive interference already came with a large effect size ($d=0.92$), but the effect size for the significant difference between sleep and wake groups in the presence of retroactive interference was even larger ($d=3.07$). We followed Lakens (2013) to derive the effect size for the significant interaction reported by Ellenbogen et al. (2006a), and indeed, with a partial eta squared of .11 ($f=0.35$), it can be interpreted as medium to large in size. Evaluating the original effect size on the basis of a replication study with a larger sample size could also help to better understand the so far mixed state of the literature.

## Method

The data collection for this study was carried out in 2021, and thus in a time during which our lives were heavily affected by the Covid-19 pandemic. Conditions at our university did not allow in-person testing in the lab, which is why we decided to collect data in an online format instead (i.e., via Zoom sessions rather than in the lab; see below for further procedural details). This constitutes a deviation from Ellenbogen et al. (2006a), who exclusively collected data in a lab environment. Such a deviation is of course not ideal for a replication study, but since the only other option was to cancel the study entirely, we decided to collect data nonetheless. On the positive side, Zoom meetings still enable relatively natural interactions between participant and experimenter, thus mimicking lab testing as closely as possible. In addition, the important sleep/wake intervals still remained matched across studies (since participants in the Ellenbogen et al. study also spent these intervals outside of the lab, in their own personal environments).

### Participants

In determining sample size, we had to balance our goal to substantially increase statistical power with the feasibility of conducting the actual study within a reasonable time frame. We therefore estimated the maximum number of subjects that we might be able to test and used this number to run a sensitivity analysis. This analysis suggested that 30 subjects per condition (total n=120) would enable us to detect medium-sized effects of $f=0.26$ in a 2 x 2 between-subjects ANOVA ($1-beta =.80$, $alpha =.05$), which is well below the effect size that was evident in Ellenbogen et al. (2006a).

Subjects were recruited from the student population at Regensburg University and received partial course credit for participating. We applied the same exclusion criteria as Ellenbogen et al. (2006a). The data of 14 participants had to be discarded because their Epworth Sleepiness Scale score was > 10 (see Johns, 1991). One more participant had to be excluded due to habitual sleep duration of less than 6 h. We also monitored for habitual sleep onset after 2 a.m., known sleep disorders, neurological diseases, the use of medication or illegal drugs, as well as for compliance with instructions during the delay (i.e., whether participants stayed awake during the day or slept regularly during the night), but no exclusions were necessary based on these criteria. In sum, the data of 15 participants were excluded. We recruited and tested 15 new participants to replace the excluded data sets.

The final sample consisted of 120 healthy participants (81 women, 39 men), with age ranging between 18 to 34 years ($M = 22.73$, $SD = 3.02$). Subjects in the sleep conditions reported to have slept regularly between the two experimental sessions ($M = 7.95 h$, $SD = 0.82$). Subjects in the wake conditions remained awake between sessions and reported no daytime naps.

### Design

Following Ellenbogen et al. (2006a), the experiment applied a 2 (12-h sleep vs. 12-h wakefulness) x 2 (with vs. without additional learning) between-subjects design^1^. Half of all participants began the study at 9 p.m. After studying paired associates, they were asked to sleep regularly during the night and to return for a second session the next day at 9 a.m., after a 12-h delay. The other half of participants began the study at 9 a.m. After studying the same paired associates, they were asked to stay awake during the day and to return for a second session the same day at 9 p.m., after a 12-h delay. In addition, during the final test after the 12-h delay, half of all participants in both delay conditions simply were asked to recall the studied paired associates (without additional learning). The other half of participants was however asked to study additional word pairs to elicit retroactive interference, and then completed the final memory test.

### Material

The word pair materials used in Ellenbogen et al. (2006a) were kindly made available to us by the second author of the study (Dr. Justin Hulbert). For parts of these materials, however, translations into German resulted in awkwardly long words, as well as words that may not be very commonly used in German. We therefore decided against using the (translated) original materials in our study. Instead, we created a new set of materials, following the same compilation criteria as described in Ellenbogen et al. (2006a). 60 nouns were drawn from the Toronto Word Pool (Friendly et al., 1982), matched for imageability, frequency, and concreteness. The nouns were chosen such that their German translations comprised two syllables and were commonly used words. The 60 words were then randomly split into three sets of 20 items (forming the A, B, and C lists). Words from the A list were paired with words from the B and C lists to create two lists of paired associates (i.e., A-B and A-C). If random pairing created word pairs with a clear semantic association, these word pairs were rerandomized. As in the original study, item assignments to list B or C were counterbalanced across participants (i.e., A-B and A-C lists were equally often used as materials in the first and second study sessions, respectively). Stimulus materials and data for this project are available on the Open Science Framework (https://osf.io/xvuq9/).

Additionally, the Epworth Sleepiness Scale (Johns, 1991) was used to assess pathological sleepiness. The sequential finger tapping task served as a distractor task (Karni et al., 1998; Walker et al., 2002).

### Procedure

*First session.* Subjects participated from their homes and received an invitation for a Zoom meeting via e-mail after signing up for the study. They were greeted by an experimenter, who provided basic information about the study as well as participants’ rights and asked for verbal consent to participate in the study. All subjects agreed to participate. The experimenter kept their camera and microphone activated during the Zoom meeting to facilitate communication, and subjects were asked to do the same. Importantly, no video or audio recordings were made to protect subjects’ privacy; this aspect was also emphasized to participants. For task and stimulus presentation, the experimenter shared their desktop via screensharing and participants were given remote control to enter their responses.

To start off, participants were asked to provide demographic information about themselves and to fill out the Epworth Sleepiness Scale. Next, participants studied 20 paired associates. Encoding closely followed Ellenbogen et al. (2006a) and comprised two phases. In an initial study-only phase, the 20 paired associates were presented back to back, in a fixed order across subjects, and for 7 sec each. Word pairs were presented in capital letters and a black font, centered on a white screen.

In the second encoding phase, the list was presented again, one pair at a time and in the same order, but now an anticipation-plus-study procedure was applied (see also Bower et al., 1994). The first word of each pair was presented on the screen and subjects were asked to type in the second word. Immediate feedback was then presented for 2 sec (either: “Correct. The correct pairing is:” or “Incorrect. The correct pairing is:”) before the next trial started. The list was tested repeatedly in this manner, and word pairs were dropped from further repetitions after they were correctly recalled three times. This procedure continued until all pairs were dropped from the list. Thus, there were at least three repetitions for each word pair and the learning criterion was set to 100% correct for all participants.

With this, the first experimental session was completed, and participants were asked to return to the Zoom meting for a second session after a delay of 12 h (at 9 a.m. or p.m. for the sleep and wake groups, respectively).

*Second session.* After the 12-h delay, following Ellenbogen et al. (2006a), participants in the sleep conditions were asked to indicate when they went to bed and how long they slept, whereas participants in the wake conditions were asked to report if they took any naps during the day. After these self reports, participants in conditions with additional learning were asked to study a new list of 20 interfering (A-C) word pairs. The same two-phase encoding procedure was applied as with the original (A-B) word pairs. After learning the interfering word pairs, participants performed a 12-min finger-tapping task before moving on to the final test (see also Ellenbogen et al., 2006a).

In Ellenbogen et al. (2006a), participants in no-additional learning conditions were tested immediately after the 12-h delay and their self reports, without any distractor task. This creates a confound, however, because, under such conditions, the retention interval before the final test is considerably longer in the presence than in the absence of additional learning. Indeed, it could be argued that this confound may increase potential interference effects (due to longer retention intervals in the conditions with additional learning). Yet, because the present study was a replication study and because our goal was to stay as close to the original study as possible, we followed the same procedures as those used by Ellenbogen et al. (2006a). Participants in conditions without additional learning were thus tested immediately after the 12-h delay interval and their self reports.

At test, participants were provided with a list of all 20 A-list stimuli (i.e., the A words of the A-B and A-C pairs), and were asked to recall the word(s) that complemented each pair. They were given 6 min to complete this recall task. Although the outcome of interest was recall of the A-B word pairs, participants in the additional learning conditions were asked to simultaneously record the A-C word pairs during testing, and to indicate which list each response belonged to (A-B if learned before the delay or A-C if learned after the delay). This corresponds to a modified modified free recall (e.g., Barnes and Underwood, 1959), which supposedly eliminates competition between interfering response options. Consistent with Ellenbogen et al. (2006a), only those words that were recalled, identified with the correct cue word (A) and ascribed to the correct list were counted as correct responses. When the memory test was completed, participants were debriefed, thanked and compensated for their participation.

**Fig. 1:**
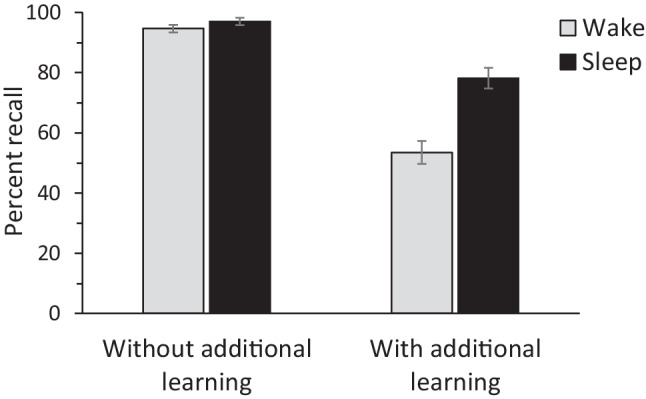
Mean recall of the A-B list in percent, shown as a function of delay (wakefulness vs. sleep) and additional learning (absent vs. present). Error bars indicate ±1 standard errors of the mean

## Results

### Main Analyses

Following Ellenbogen et al. (2006a), the A-B recall data were arcsine transformed before data analysis. A 2 x 2 ANOVA showed a significant main effect of delay, $F(1, 116) = 26.44$, $MSE = 0.05$, $p <.001$, $\eta ^2 =.19$, reflecting better recall after sleep than wakefulness (87.58% vs. 74.08%). Moreover, there was a significant main effect of additional learning, $F(1, 116) = 140.26$, $MSE = 0.05$, $p <.001$, $\eta ^2 =.55$, indicating better recall in the absence than in the presence of additional learning (95.83% vs. 65.83%). Most critically, there was also a significant interaction between the two factors, $F(1, 116) = 9.86$, $MSE = 0.05$, $p =.002$, $\eta ^2 =.08$, suggesting that the influence of additional learning depended on type of delay (see also Fig. 1). Higher recall after sleep compared to wakefulness was found in the presence of additional learning (78.17% vs. 53.50%), $t(58) = 4.95$, $p <.001$, $d = 1.28$, 95% CI [0.72, 1.83], but not in its absence (97.00% vs. 94.67%), $t(58) = 1.82$, $p =.073$, $d = 0.47$, 95% CI [$-$0.04, 0.98]. Additional learning impaired recall, but this effect was more pronounced after wakefulness, $t(58) = 11.08$, $p <.001$, $d = 2.86$, 95% CI [2.13, 3.58], than after sleep, $t(46.66) = 5.91$, $p <.001$, $d = 1.53$, 95% CI [0.94, 2.10].^2^

The above significance tests can be complemented by using the Bayesian information criterion to compute posterior probabilities for the null and alternative hypotheses being correct given the observed data (D; see Masson, 2011, for details). In the absence of additional learning, the resulting posterior probabilities for sleep-associated benefits were P$_{BIC}$(H$_{0}$ $\vert$D) = 0.594 and P$_{BIC}$(H$_{1}$ $\vert$D) = 0.406, which provides weak evidence in favor of the null hypothesis. In the presence of additional learning, however, the resulting posterior probabilities for sleep-associated benefits were P$_{BIC}$(H$_{0}$ $\vert$D) = 0.0002 and P$_{BIC}$(H$_{1}$ $\vert$D) = 0.9998, which provides very strong evidence in favor of the alternative hypothesis. These analyses are consistent with the significant interaction effect reported above, suggesting a stronger benefit of sleep in the presence of additional learning.

### Additional Analyses

Ellenbogen et al. (2006a) reported two additional analyses. The first analysis addresses potential time-of-day effects. Because sleep groups start the experiment at 9 p.m., but wake groups at 9 a.m., differences could be due to circadian influences (rather than memory consolidation). To address this, number of trials needed to reach the 100% learning criterion during encoding was examined. For the A-B list encoded in the first session, there was no significant difference between sleep conditions ($M = 89.88$, $SD = 31.26$) and wake conditions ($M = 93.65$, $SD = 29.90$), $t(118) = -0.67$, $p =.501$, $d = -0.12$, 95% CI [$-$0.48, 0.24]. For the A-C list encoded in the second session, mean number of learning trials seemed lower in the sleep condition ($M = 75.67$, $SD = 13.89$) than in the wake condition ($M = 87.87$, $SD = 30.86$), but the difference was also not significant, $t(40.29) = -1.98$, $p =.055$, $d = -0.51$, 95% CI [$-$1.02, 0.01].

The second additional analysis concerns recall of the A-C list, used to elicit retroactive interference. As the A-B list, the data were arcsine transformed prior to analysis. Consistent with Ellenbogen et al. (2006a), there was no significant difference between the sleep and wake condition (96.33% vs. 97.33%), $t(58) = 0.34$, $p =.736$, $d = 0.09$, 95% CI [$-$0.42, 0.59]. The high recall rates for the A-C list additionally suggest that the main results for the A-B list do not reflect a problem of list confusion (i.e., participants did not systematically misattribute items from the A-B list to the A-C list).

## Discussion

The results replicate Ellenbogen et al. (2006a). Additional learning after 12 h reduced memory for the initially studied A-B list, but this retroactive interference was significantly reduced after sleep relative to wakefulness. The replication was successful despite some differences to the original study. Due to Covid-19, our study was run online, whereas Ellenbogen et al.’s (2006a) study was run in the lab. Moreover, we adapted study materials for use in German. University students received partial course credit for participating, whereas participants in Ellenbogen et al. (2006a) received payment. Considering these differences, the similarity of results is striking, although there were some numerical differences (e.g., performance in our wake conditions was higher relative to Ellenbogen et al., 2006a).

### Relation to previous work: effect sizes and statistical power

The Ellenbogen et al. (2006a) findings were highly influential in suggesting that sleep plays more than a passive role for memory consolidation, but the orginal results were never replicated with a full between-subjects design. Overall, the results of subsequent studies were mixed, which was our main motivation for trying to replicate the original study. How can the present findings be integrated into this literature?

Our results show that the original findings by Ellenbogen et al. (2006a) can be replicated. Applying the same procedure, but a sample that was 2.5 times larger, the findings seem reliable and robust. The critical interaction between delay and additional learning was medium to large in size in the original study ($\eta ^2=.11$, $f=0.35$), and we observed a similar effect size in our study ($\eta ^2=.08$, $f=0.29$). Our data thus suggest no major overestimation of the effect size associated with the critical interaction. The case is however different for single comparisons. The benefit of sleep relative to wakefulness in the presence of additional learning was very large in Ellenbogen et al. (2006a; $d=3.07$). In comparison, this effect was substantially reduced in our study, though still large ($d=1.28$). Ellenbogen et al. (2006a) only reported effect sizes for single comparisons, not for the critical interaction effect. Inasmuch as subsequent studies used this directly available effect size for determining samples, insufficient statistical power may have been the consequence.

We ran power analyses to look into this issue. For between-subjects designs, we assumed an effect size of $f=0.32$ for the critical interaction (the average of the effect sizes in Ellenbogen et al., 2006a, and the present replication). Samples of 79 participants provide 80% power to detect interaction effects of this size, whereas samples of 105 participants provide 90% power. A glance at Table 1 shows that, apart from the present study, only two previous studies had sufficient power (Bäuml et al., 2014; Schoch et al., 2017), and both found no significant interaction effect. Study materials in both studies were however quite different from those used here and in Ellenbogen et al. (2006a; Bäuml et al., 2014: semantically categorized lists; Schoch et al., 2017: neutral/emotional pictures).

For mixed designs, with interference as within-subject factor, we assumed an effect size of $f=0.20$ for the critical interaction (the average of the effect sizes in Ellenbogen et al., 2009, and the direct replication by Bailes et al., 2020). Here, samples of 52 participants provide 80% power to detect such effects, whereas samples of 68 participants provide 90% power. Consulting Table 1, only the replication study by Bailes et al. (2020) had sufficient power to defect interaction effects of this size – all other sample sizes were too small.

The above power analyses are sobering. Considering this state of the literature in addition to the present work, it is still unclear whether sleep protects memories from retroactive interference.

### Moving forward

A constructive way to gain an answer to this theoretically relevant question in future work may be to switch gears and pursue a collaborative approach, for instance in the context of a multilab replication study. Following this approach, single labs would not work in isolation, but contribute data to a larger project, addressing the issue a bit more broadly (e.g., with some built-in experimental variation) and with higher statistical power.

In this context, three study-design features seem like important candidates for possible adjustment. The first concerns the potential influence of ceiling effects. With learning criteria of 100% correct, high recall levels may mask sleep benefits in the absence of additional learning. Such ceiling effects could in principle explain the significant interaction effects observed in Ellenbogen et al. (2006a) as well as in the present replication study. Indeed, some previous studies that did not suffer from ceiling effects found no evidence for a protective role of sleep (see Bailes et al., 2020; Bäuml et al., 2014). The second issue concerns the confound between the induction of retroactive interference and the length of the retention interval. In Ellenbogen et al. (2006a) as well as in the present study, participants in conditions without additional learning were tested immediately after the sleep/wake interval, whereas additional learning needed time, resulting in differences in the length of the retention interval. Better matching delays across conditions would eliminate this confound. So far, it seems that delay may only have been adequately matched in Bäuml et al. (2014), who found no protective influence of sleep. The third issue concerns better controls for time-of-day effects (e.g., a 24-h p.m. control condition with additional learning, as used in Ellenbogen et al., 2006a). Time-of-day effects based on number of learning trials were not significant in the present study, but since our sample size was limited, too, smaller (but nevertheless meaningful) effects may simply not have been detected.

A collaborative replication effort may address all these potentially critical issues, while holding other study-design features constant that have varied somewhat across prior work (e.g., type of study material, ranging from word pairs to spatial locations, pictures, and sounds in previous studies). Such an endeavor would need to be an orchestrated effort, but if successful, could result in a large data set that is able to provide a straightforward answer to the theoretically important question of whether sleep protects memories from interference (and under what conditions).

## Data Availability

Materials and data are available on the Open Science Framework (https://osf.io/xvuq9/).

## Footnotes

1 The study by Ellenbogen et al. (2006a) included an additional 24-h condition to evaluate time-of-day effects. Because the present study was concerned with replicability of the study’s core finding, this additional control condition was not part of the replication attempt.

2 We reran the analyses with percentage correct as the dependent measure. The pattern of results did however not change. The critical interaction between delay and additional learning remained intact, $F(1, 116) = 16.75$, $MSE = 223.35$, $p <.001$, $\eta ^2 =.13$. A benefit of sleep was found in the presence of additional learning, $t(58) = 4.78$, $p <.001$, $d = 1.23$, 95% CI [0.68, 1.78], but not in its absence, $t(58) = 1.32$, $p =.193$, $d = 0.34$, 95% CI [$-$0.17, 0.85]. Additional learning impaired recall after sleep, $t(36.18) = 5.19$, $p <.001$, $d = 1.34$, 95% CI [0.77, 1.90], but more so after wakefulness, $t(35.37) = 10.10$, $p <.001$, $d = 2.61$, 95% CI [1.91, 3.30].
